# Effect of Breed and Gender on Meat Quality of *M. longissimus thoracis et lumborum* Muscle from Crossbred Beef Bulls and Steers

**DOI:** 10.3390/foods8050173

**Published:** 2019-05-21

**Authors:** Jamie Cafferky, Ruth M. Hamill, Paul Allen, John V. O’Doherty, Andrew Cromie, Torres Sweeney

**Affiliations:** 1 Department of Food Quality and Sensory Science, Teagasc Food Research Centre, Ashtown, 15 Dublin, Ireland; jamie.cafferky@teagasc.ie (J.C.); Ruth.Hamill@teagasc.ie (R.M.H.); paul.allen869@gmail.com (P.A.); 2 School of Veterinary Medicine, University College Dublin, Belfield, 4 Dublin, Ireland; 3 School of Agriculture & Food Science, University College Dublin, Belfield, 4 Dublin, Ireland; john.vodoherty@ucd.ie; 4 Irish Cattle Breeding Federation, Shinagh House, Bandon, P72 X050 Co. Cork, Ireland; acromie@icbf.com

**Keywords:** beef quality, castration, breed, shear force, intramuscular fat

## Abstract

The objective of this study was to determine whether sire breed and/or castration had an effect on meat quality of *M. longissimus thoracis et lumborum* (*LTL*) muscle from crossbred bulls and steers and to investigate the relationship amongst the traits examined. Warner–Bratzler shear force (WBSF), intramuscular fat (IMF)%, cook-loss%, drip-loss%, colour (L*, a*, b*) and ultimate pH (upH) were determined in the *LTL* muscle from eight beef sire breeds representative of the Irish herd (Aberdeen Angus, Belgian Blue, Charolais, Hereford, Limousin, Parthenaise, Salers and Simmental). The results indicate that IMF%, cook-loss% and drip-loss% were associated with breed (*p* < 0.05); while WBSF, IMF% and cook-loss% differ between genders (*p* < 0.05). Steer *LTL* had a greater IMF% and exhibited reduced WBSF and cook-loss% in comparison to the bull *LTL* (*p* < 0.05). This study provides greater insight into how quality traits in beef are influenced by breed and gender and will support the industry to produce beef with consistent eating quality.

## 1. Introduction

Factors such as breed, gender, age of animal at slaughter, diet and feeding regime can influence muscle characteristics, which in turn affect meat quality [1,2,3]. Meat quality attributes such as tenderness, colour, flavour, juiciness, and water-holding capacity (WHC)-related traits—cook-loss% and drip-loss%—influence consumer satisfaction [4,5]. The relative importance and value of particular meat quality traits vary according to the type of meat product being produced and marketed and also the end target consumer of the product; e.g., tenderness is more important for beef meat than sheep meat [5]. As progress is made on the development of quality traits, their relative value is also altered, which has potential to impact on their prioritisation in animal breeding programmes [6]. In order to meet the demand for the high-quality product anticipated by consumers, beef producers must focus on improving the quality in addition to quantity [7]. This has increased the focus of both industry and academia on husbandry and breeding strategies aimed at improving meat eating quality traits [5].

Growth rate, carcass yield, feed efficiency and carbon efficiency are positively influenced in bulls (intact adult males) compared to steers (castrated adult males) [8,9]. Carcass fat is, however, a limiting factor for bull beef production [10]. Gender has been associated with many aspects of meat quality and has been proposed to favourably influence fat deposition and tenderness [11]. Steers are commonly used to produce the highest quality beef that is distinguished from beef attained from bulls and obtains a premium price in restaurants and markets in developed nations [11].

Breed also influences meat quality. Individual cattle breeds have been developed through extensive long-term selection for specific production attributes, such as increased growth rate, carcass conformation and intramuscular fat (IMF) [12,13]. Early maturing breeds such as Angus and Hereford have higher levels of IMF (and associated traits tenderness and flavour) in comparison to late maturing continental breeds [14,15,16,17] and this is reflected in the price [18].

Pre-slaughter handling and subsequent post-mortem processing play a major role in the final quality attributes of meat. Factors such as feed withdrawal [19], transport time [11] and stress during transport [20] can have a negative impact on subsequent meat quality. Bulls are more sensitive than steers to all these factors due to their sexual maturity and greater aggression in the lairage. This can lead to higher ultimate pH measurements and unfavourable meat quality [11,21,22]. 

Gaining insights into how meat quality traits are affected by animal breed and gender could inform pre-slaughter handling practises and post-mortem technologies aimed at maximising quality [22]. Furthermore, it could allow meat processors to optimise meat management systems based on specific quality traits (due to the animal’s breed or gender) [23,24,25]. Therefore, the objective of this study was to determine whether sire breed and/or castration had an effect on the meat quality of *M. longissimus thoracis et lumborum* (*LTL*) muscle from crossbred bulls and steers and to investigate the relationship amongst the traits examined. 

## 2. Materials and Methods 

### 2.1. Animals and Sample Preparation

Crossbred bull and steer progeny were obtained and reared under the same feeding and environmental conditions by the Irish Cattle Breeders Federation Tully Progeny Test Centre (Tully, Kildare, Republic of Ireland). Bull and steer progeny examined in this study were bred from crossbred commercial suckler dams artificially inseminated by elite Irish beef breed bulls. Animals were acclimatised for approximately 30 days before starting a 90-day testing period. Bulls were offered an *ad-libitum* concentrate diet with 3 kilograms (kg) of fresh hay, while steers were offered 8 kg of concentrates and 5 kg of hay on a fresh-weight basis per head per day. Hay was offered to support the healthy functioning of the rumen and to reflect an Irish commercial high concentrate-based dietary regimen. All animals were finished to a specified carcass conformation and fat score range. For bulls this was U− to E+ conformation score, 3− to 5= fat score and 678 kg live weight (±58 kg); for steers this was R− to E= conformation score, 2+ to 5+ fat score and 637 kg live weight (±64 kg). Bulls were slaughtered at approximately 487 days old (±24 days), while steers were slaughtered at approximately 634 days old (±52 days). Eight beef breeds, with numbers representative of that in the Irish herd, were included as part of this study as follows: Aberdeen Angus (AA; bull *n* = 36, steer *n* = 28), Belgian Blue (BB; bull *n* = 67, steer *n* = 10), Charolais (CH; bull *n* = 127, steer *n* = 41), Hereford (HE; bull *n* = 2, steer *n* = 11), Limousin (LM; bull *n* = 234, steer *n* = 62), Parthenaise (PT; bull *n* = 11, steer = 4), Salers (SA; bull *n* = 25, steer *n* = 16) and Simmental (SI; bull *n* = 63, steer *n* = 16). Animals were slaughtered in batches of approximately 50, between February 2014 and May 2017 in a commercial plant by electrical stunning (50 Hz) followed by exsanguination from the jugular vein. Between 40–60 min post exsanguination, carcasses were split in half then chilled for 24 h at 2 °C. Twelve steaks with a thickness of 2.54 cm were removed sequentially from the right-side *LTL* 48 h post-mortem starting at the rump end and vacuum packaged. Steaks were labelled 1–12 according to the trait being measured in order to ensure the analysis was conducted in a consistent location within the *LTL* muscle. Steaks were frozen at −20 °C after 2 or 14 days of ageing at 4 °C, dependent on the trait being determined.

### 2.2. Warner–Bratzler Shear Force and Cook-Loss%

For the determination of cook-loss%, 14-day aged steaks were frozen at −20 °C until analysis then thawed within unsealed plastic vacuum bags in a circulating water bath at room temperature (20 °C). Steaks were trimmed of external fat, blotted lightly with tissue paper to remove moisture and weighed. Steaks were immersed in a water bath for cooking (Grant Instruments Ltd., Royston, England) at 72 °C until an internal core temperature of 70 °C was reached using a temperature probe (Eirelec Ltd., Dublin, Ireland). Samples were cooled to room temperature, blotted lightly with tissue paper and weight was recorded. Samples were then placed within new unsealed vacuum bags and left to temper overnight in a fridge at 4 °C. Cook-loss was expressed as a percentage of the raw weight of the steak as follows:

Cook-loss (%) = (raw weight − cooked weight)/raw weight × 100(1)

Following cook-loss determination, the tempered steaks were used for Warner–Bratzler shear force analysis according to a modified version of American Meat Science Association guidelines [26]. Seven cores per steak were removed for analysis using a 1.27-cm core and sheared perpendicular to the fibre direction using the Instron 4464 Universal testing machine (Instron Ltd., Buckinghamshire, UK), with a load cell of 500 N and a cross head speed of 50 mm/min, and analysed using Bluehill^®^2 Software (Instron Ltd., Buckinghamshire, UK). The maximum peak force recorded during analysis was reported as Newton (N) shear force. The highest and lowest measurements were excluded with the average of the remaining 5 cores recorded as the result to reduce standard deviation.

### 2.3. Intramuscular Fat% 

IMF% was determined on 2-day aged steaks using the Smart System-5 microwave moisture drying oven and NMR Smart-Trac rapid fat analyser (CEM Corporation, Matthews, NC, USA) using AOAC Official Method 985.14 [27]. In brief, steaks previously frozen at −20 °C were thawed within unsealed plastic vacuum bags in a circulating water bath at room temperature (20 °C). Once thawed, the steaks were trimmed of external fat, cut into cubes approximately 2.5 × 2.5 cm and placed into a RobotCoupe R2 blender and homogenised to a fine consistency. Two grams of homogenised meat free of connective tissue was then placed within the Smart-Trac for analysis.

### 2.4. Ultimate pH 

Ultimate pH (upH) measurements were collected from carcasses 48 h post-mortem by placing a calibrated pH meter (Hanna HI 9125 pH meter, Woonsocket, RI, USA) within the loin between the 12th and 13th rib avoiding bone and connective tissue. Calibration of the pH electrode was performed with standardized buffers (pH 4.0 and 7.0).

### 2.5. Drip-Loss%

Drip-loss% was analysed according to the procedure of Honikel and Hamm [28]. From each steak (2-day aged samples, assigned for drip-loss analysis), a piece was removed (2.5 cm in thickness, 7.5 cm in length and 5 cm in width) avoiding connective tissue and large areas of fat not representative of the sample. Samples for drip-loss weighed approximately 100 ± 5 g and were lightly blotted with tissue paper, weighed, then suspended by string and an unfolded paperclip (formed into a hook shape) within an expanded clear plastic bag, with care taken to ensure the sample did not come into contact with the bag. The samples were suspended in a chill room at 4 °C for 96 h, after which, the surface was lightly blotted with a tissue and re-weighed. Drip-loss was expressed as a percentage of the original weight of the steak as follows:

Drip-loss (%) = (initial weight − final weight)/initial weight × 100.(2)

### 2.6. Colour

Colour measurements were performed using the HunterLab UltraScan Pro CIE L*a*b* system with a dual beam xenon flash spectrophotometer (Hunter Associates Laboratory, Inc., Reston, VA, USA). CIE L* (lightness), a* (redness), b* (yellowness) values were recorded. The illuminant (D65, 10°) consisted of an 8° viewing angle and a 9.9-mm port size. Calibration was carried out using a white standard tile (*L* = 100) and light trap (*L* = 0). The white tile was covered in cling film prior to calibration to prevent any effect on the colour reading. Steaks (2 d post-mortem) were wrapped in cling film and allowed to bloom for 1 h prior to measurement. Three measurements were taken in three separate locations on each steak, avoiding intramuscular fat and connective tissue.

### 2.7. Statistical Analysis

Statistical analysis was performed by two-way Analysis of Variance (ANOVA) using Tukey–Kramer adjusted generalised linear model (GLM) procedures of Statistical analysis Software (SAS) 9.4 (SAS Institute, Cary, NC, USA). Pearson Correlations between beef *LTL* quality attributes were calculated using the CORR procedure in SAS. Differences were considered significant at the *p* < 0.05 level.

## 3. Results

### 3.1. Correlations between Traits

Pearson correlations between eight beef quality traits examined as part of this study are presented in Table 1. Cook loss, drip loss, pH and colour traits showed a number of correlations with each other. WBSF and IMF were negatively correlated, with higher fat-content meat being associated with lower shear force. Cook loss was also linked to both fat content and shear force.

### 3.2. Effect of Breed and Gender on Meat Quality

The effects of breed and gender on *LTL* muscle quality traits from eight beef breeds are presented in Table 2. Gender had a significant effect on WBSF values, with bull *LTL* samples having higher shear force than that of steer *LTL*. However, no effect was observed on WBSF values for breed.

IMF was associated with both breed and gender, with *LTL* from steers containing almost twice the IMF% of bull *LTL* (2.85 and 1.27%, respectively; Table 2). AA sired *LTL* samples had the highest levels of IMF in the current dataset (2.78%) with BB and PT (1.12%) sired progeny, the lowest. Cook-loss was significant for both breed and gender effects, with LM-sired progeny having the lowest cook-loss values (29.09%). Bull *LTL* cook-loss% was higher than that of steers (30.4 and 29.25%, respectively). Drip-loss values only tended to be higher in steers than in bulls (3.41 and 2.73%); however, sire breed had an effect on drip-loss% values. The AA sire progeny had the lowest drip-loss% measured (2.15%), with BB having the highest (4.11%).

## 4. Discussion

This study illustrates the significant effect castration has on important technological beef quality traits, such as increasing IMF%, reducing WBSF and improving cook-loss%. Moreover, the quality attributes, IMF, cook-loss and drip-loss, varied significantly according to sire breed. However, very little variability exists for texture, as reflected by WBSF scores, and sire breed was not determined to be a significant factor for this trait.

Interesting correlations amongst the traits examined as part of our study were found. Notably, the three traits affected by gender, WBSF, IMF% and cook-loss%, were all significantly correlated to each other. In this study, traits relating to different aspects of meat quality show trends that were associated with each other, pointing to particular animals having an overall higher quality, relative to others within the study. For example, animals with higher IMF% and redness values had lower WBSF scores and reduced cook-loss%, which could be considered to be a combination of trait values associated with good overall meat quality. Monteiro et al. [29] also reported a positive relationship between WBSF and cook-loss%. They concluded that cook-loss% influenced WBSF more than any other physiochemical trait and was the reason for shear force variation. The multifactorial relationship amongst these traits suggests that selecting one of these correlated traits for improvement may have a beneficial effect on other meat quality attributes. The two water-holding capacity related traits (cook-loss% and drip-loss%) were not correlated with each other. The two traits capture different aspects of fluid loss in processing. Drip-loss is reflective of exudate, consisting mainly of water and proteins, whereas cook-loss may also be associated with glycogen potential and additionally melting fat during the thermal processing [30]. These traits have been well studied in pork [31]. Interestingly, a comparable lack of correlation between these traits is present in beef in the current study.

The highest scoring sire breed for IMF% was the early-maturing AA sire offspring, which was significantly different from the two lowest scoring sire breeds for this trait, i.e., the late maturing continental BB and PT sire progeny. In a comprehensive study by Gagaoua et al. [1], breed had a significant influence on IMF%, with AA bulls having twice the IMF% relative to continental breed bulls examined in their study. Differences in IMF% values between breeds is mostly attributed to genetics, with early maturing breeds (such as AA and HE) having higher fat deposition than continental breed animals [16]. Steers had greater than twice the IMF% relative to bulls, consistent with Moran et al. [10] and Nian et al. [32] who reported that gender was significant for IMF. The difference between IMF% values between bulls and steers is attributed to the removal of the testes in steers, which arrests sexual maturation leading to reductions in growth rate and muscular development and increases fat deposition and accelerates the fattening period [33]. Other hormones involved in muscle and adipose tissue metabolism include leptin, growth hormone, insulin, cortisol, insulin-like growth factor 1, thyroxin and triiodothyronine [34,35,36]. Testosterone binds to receptors within the muscle, increasing the incorporation of amino acids into protein and increasing the capacity for muscular development and growth rate [37]. This results in an increase in muscle mass without increases in IMF% [37]. Greater IMF values in steers are attributed to the diminished physiological effects of this androgen, reducing plasma lipids, increasing lipolysis by adipocytes and stimulating androgen receptors [38,39]. The castration of bulls is directly involved in the upregulation of the lipogenic gene expression of fatty acid synthase (FASN) and acetyl-CoA carboxylase (ACC); furthermore, it also downregulates the lipolytic gene expression of monoglyceride lipase (MGL) and adipose triglyceride lipase (ATGL) [40]. Hence, castration contributes to improved IMF deposition mediated through increased lipid uptake and lipogenesis and decreased lipolysis [40].

WBSF values exhibited a gender effect but not a breed effect, with steers exhibiting a WBSF value approximately 7 N lower than bulls, in agreement with Nian et al. [32]. A gender effect for WBSF between bulls and steers was also found by Mberema et al. [41]. However, they observed 14-day aged bull *LTL* to be more tender than steer and heifer *LTL*. The results of the current study contrast with Moran et al. [10] who found no effect on WBSF values between bulls and steers. Gerrard et al. [42] reported that any difference in WBSF values explained by gender is not observed following 13 days of ageing. However, the current study aged samples for 14 days and an effect was still observed. Other factors that contribute to the textural toughness of beef (namely sarcomere length and collagen) were not assessed but may play a role in the differences observed between genders, with bulls having shorter sarcomeres and higher levels of collagen within muscle [32]. Various studies on the use of late maturing beef bulls have also reported that breed has no significant effect on instrumental tenderness [13,43,44]. With regards to breed effect and sheer force, Marino et al. [45] reported significance when comparing three cattle breeds contrasting in traditional breed purpose (Friesian dairy bulls; Romagnola crossbred beef bulls; Podolian bulls—indigenous to southern Italy), with the indigenous Podolian bulls significantly tougher than their counterparts. This is in contrast to the findings of the current study as sire breed had no effect on shear force values.

In the current study, cook-loss% is associated with both breed and gender. The breed effect was observed between LM and SI sire breeds. This is in agreement with Chambaz et al. [14] who also observed a significant difference in cook-loss between these two breeds. IMF is one of the factors associated with cook-loss; as IMF% increases, cook-loss% values decrease [46]. Findings relating breed to cook-loss are sometimes conflicting. Mandell et al. [47] examined HE and SI breeds for cook-loss% and found no significance; however, when comparing AA, BB and LM bulls for cook-loss, Cuvelier et al. [2] found AA bulls to be significantly different. Steers had lower cook-loss% than the bulls examined which is in agreement with numerous studies [10,32,47]. The differences observed between bulls and steers may be attributed to the larger muscle fibre diameter of bulls, induced by androgens, with muscles of increased cross-sectional area exhibiting greater cook-loss [9,48]. In contrast, Knight et al. [49] found no gender effect between bulls and steers for cook-loss%. In our study, drip-loss% was significant for breed but not gender. Early maturing breeds (AA and HE) exhibited lower drip-loss% values in comparison to the larger, late maturing continental breeds such as Belgian Blue, Charolais and Parthenaise animals, indicating early maturing breeds have the potential to have juicier meat and less reduction in yield associated with hanging. Similar trends regarding drip-loss values and breed effect were shown by Cuvelier et al. [2] when studying BB, LM and AA breeds, and Chambaz et al. [14] when studying AA, LM, SI and CH breeds. Cuvelier et al. [2] suggested that higher drip-loss% values in larger animal breeds (such as BB and PT, the highest scoring for this trait) may be due to their higher meat:water content. The lower collagen content of double muscle animals may also contribute to increased drip-loss, as WHC is known to increase with increasing amounts of connective tissue [50]. Breeds exhibiting lower drip-loss values have the added economic benefit of less product weight loss, leading to higher financial gain on carcass and primal cuts. 

Lightness (L*), redness (a*) and yellowness (b*) values were not influenced by breed or gender in our study. This is consistent with Moran et al. [10] who did not see any difference when comparing colour values between bulls and steers. Nian et al. [32] also found no significant effect for gender on L* values; however, in that study, steers had significantly higher a* and b* values in comparison to bulls, which is in contrast with the current findings. Papaleo Mazzucco et al. [16] found breed to have no effect on L*, a* or b* values when comparing AA and HE steers. However, Cuvelier et al. [2] found breed to influence both L* and a* values when comparing AA, BB and LM bulls; with BB bulls having lighter and less red *LTL*, and AA bulls having the darkest and reddest *LTL* (b* values were not reported).

## 5. Conclusions

Sire breed had a significant effect on three beef quality traits analysed, IMF%, cook-loss% and drip-loss%, which were also correlated to each other. With respect to breed, Aberdeen Angus sired progeny had the highest IMF% and the lowest drip-loss%, Limousin sired offspring had the lowest cook-loss%, while Belgian Blue and Parthenaise sired progeny scored the highest for drip-loss%. Castration significantly impacted three of the beef quality traits analysed: WBSF, IMF% and cook-loss%. In comparison to bulls, steers had higher IMF% and reduced WBSF and cook-loss%, implying steer beef to be more tender and juicy, with more favourable IMF%. This study supports the hypothesis that breed and gender influence meat quality traits. 

## Figures and Tables

**Table 1:** Pearson correlation coefficients between quality traits of beef *LTL.*

	IMF (%)	upH	Cook-Loss (%)	Drip-Loss (%)	L*	a*	b*
WBSF	−0.26 ***	−0.05	0.19 ***	−0.16 ***	0.06	−0.15 ***	−0.05
IMF (%)		−0.015	−0.22 ***	−0.08	−0.07	0.1 **	0.05
upH			−0.15 ***	0.03	−0.01	−0.01	−0.01
Cook-loss (%)				0.06	0.18 ***	0.23 ***	0.16 ***
Drip-loss (%)					0.23 ***	0.04	0.13 **
*L* *						0.2 ***	0.42 ***
*a* *							0.83 ***

* *p* < 0.05, ** *p* < 0.01, *** *p* < 0.001; WBSF—Warner–Bratzler shear force; IMF—Intramuscular fat; upH—Ultimate pH.

**Table 2:** Effect of breed and gender on quality traits of *M. longissimus thoracis et lumborum* (least squares mean and standard error).

	Breed								Gender		*p*-Value	
	AA	HE	LM	CH	SA	SI	BB	PT	Bull	Steer	Breed	Gender
*n*	64	13	296	168	41	79	77	15	565	188	-	-
Trait												
WBSF	41.59 (1.2)	36.96 (3.8)	42.69 (0.7)	40.62 (0.9)	42.57 (1.6)	44.37 (1.4)	42.55 (1.7)	40.97 (2.9)	44.54 (1.2)	37.81 (1.1)	0.3122	0.0001
IMF (%)	2.78 (0.2) ^b^	2.16 (0.5) ^ab^	2.13 (0.1) ^ab^	2.05 (0.1) ^ab^	2.41 (0.2) ^ab^	2.13 (0.3) ^ab^	1.7 (0.2) ^a^	1.12 (0.4) ^a^	1.27 (0.1)	2.85 (0.1)	0.009	0.0001
upH	5.55 (0.02)	5.53 (0.05)	5.55 (0.01)	5.54 (0.01)	5.56 (0.02)	5.56 (0.02)	5.6 (0.03)	5.54 (0.04)	5.57 (0.01)	5.54 (0.01)	0.6594	0.286
Cook-loss (%)	30.15 (0.4) ^ab^	29.09 (1.1) ^ab^	29.09 (0.2) ^a^	29.66 (0.2) ^ab^	29.33 (0.5) ^ab^	30.59 (0.4) ^b^	29.63 (0.5) ^ab^	31.07 (0.8) ^b^	30.4 (0.3)	29.25 (0.3)	0.0032	0.005
Drip-loss (%)	2.15 (0.3) ^a^	2.5 (0.5) ^ab^	2.97 (0.1) ^e^	3.22 (0.1) ^f^	2.72 (0.2) ^bcd^	2.52 (0.3) ^abc^	4.37 (0.3) ^g^	4.11 (0.5) ^g^	2.73 (0.2)	3.41 (0.2)	0.0009	0.0950
L*	41.89 (0.5)	41.68 (1.1)	42.66 (0.2)	42.52 (0.3)	41.75 (0.5)	42.27 (0.7)	42.14 (0.5)	42.11 (0.9)	42.33 (0.3)	41.93 (0.3)	0.5777	0.3769
a*	14.37 (0.3)	14.15 (0.7)	14.31 (0.1)	14.56 (0.2)	14.63 (0.3)	14.27 (0.4)	13.61 (0.3)	14.17 (0.5)	14.01 (0.2)	14.52 (0.2)	0.2665	0.0635
b*	11.15 (0.3)	10.67 (0.6)	11.41 (0.1)	11.6 (0.1)	11.49 (0.3)	11.12 (0.2)	10.82 (0.3)	11.31 (0.5)	11.33 (0.2)	11.06 (0.2)	0.2902	0.3130

*n*—number of animals in each breed; AA—Angus; BB—Belgium Blue; CH—Charolais; HE—Hereford; LM—Limousin; PT—Parthenaise; SA—Salers; SI—Simmental; ()—Standard error; WBSF—Warner–Bratzler shear force; IMF—Intramuscular fat; upH—Ultimate pH; ^a,b,c,d,e,f,g^ Within a row values not sharing a common superscript are significantly different for breed.
